# Variation in Gentamicin Dosing and Monitoring in Pediatric Units across New South Wales

**DOI:** 10.1097/pq9.0000000000000015

**Published:** 2017-02-17

**Authors:** Vishal Saddi, John Preddy, Sarah Dalton, John Connors, Sarah Patterson

**Affiliations:** From the * Department of Paediatrics, Sydney Children’s Hospital, Randwick; † Discipline of Paediatrics and Child Health, University of New South Wales, Rural Medical School, Wagga Wagga; and ‡ Clinical Excellence Commission, Sydney, New South Wales, Australia.

## Abstract

**Introduction:**

Gentamicin is an aminoglycoside antibiotic with broad-spectrum bactericidal activity and is widely used in pediatric units to treat infection with susceptible organisms. This study aimed to describe the dosage regimen for gentamicin and approach to its therapeutic drug monitoring (TDM) among the pediatric units within the state of New South Wales (NSW).

**Methods:**

A questionnaire was sent electronically to representatives of 40 pediatric units in NSW, requesting details of each unit’s gentamicin dosing and TDM policy.

**Results:**

A total of 35 units responded to the survey. The majority (63%) of the units used a dose of 7.5 mg/kg of gentamicin in patients with normal renal function. More than half of the units (54%) did not have a local gentamicin dosing protocol and relied on other sources for dosing regimens. Dosing responses varied from a dose of 6 mg/kg once daily for patients more than 10 years of age to 7 mg/kg once daily on day 1, followed by 5 mg/kg once daily for patients over 10 years of age. For TDM of gentamicin, 63% of units indicated use of trough levels and 23% units used the Hartford Nomogram.

**Conclusions:**

A significant variation exists in clinical practice among pediatric units in NSW on gentamicin dosing and TDM guidelines. There is an urgent need for collaboration among nursing, medical, and pharmacy experts to achieve consensus to develop and adopt statewide uniform guidelines on gentamicin dosing and TDM.

## INTRODUCTION

A 3-year-old child was admitted to a pediatric unit with suspected urinary tract infection. The resident medical officer charted a dose of 75.6 mg of gentamicin on the medication chart; however, the nurse misread the dose as 756 mg, resulting in 10-fold overdose of gentamicin. The error was subsequently picked up 3 days later when the gentamicin level was checked. An open disclosure about the overdose was made to the family. The child developed transient gentamicin-associated acute kidney injury, resulting in multiple blood tests to monitor renal function. An outpatient audiometry testing to check for ototoxicity was organized. Though, fortunately, the child recovered completely with no long-term consequences, the parent expressed frustration over an avoidable mistake in treatment. Her words at the time of discharge from the hospital were: “Please let this incident serve as a lesson to the healthcare providers in the hospital.”

“Patients treated with aminoglycosides should be under close clinical observation because of the potential toxicity associated with their use”—a black boxed warning by the Food and Drug Administration (FDA), United States (gentamicin drug label information, first published in February 2008).

Clinical effects of gentamicin-related toxicity such as ototoxicity, nephrotoxicity, and less often neuromuscular toxicity are well known.^1^ Despite due diligence, gentamicin-related medication errors are relatively common in pediatric hospitals. In the first half of 2014, there were 30 gentamicin-related adverse events reported by the pediatric units in NSW through the Incident Information Management System *(Clinical Excellence Commission, New South Wales unpublished data, 2014*). Thus, there is limited recognition of the fact that gentamicin is one of the most common medication-related errors in the pediatric population in NSW. Bates^2^ proposed that for every medication error that harms the patient, there are up to 100 mostly undetected errors that do not. Similar concerns related to gentamicin medication safety incidents are well recognized across the world.^3–6^ There are many examples of strategies designed to improve the safety of gentamicin administration,^7–10^ yet adverse events persist.

There are 3 tertiary children’s hospitals and 40 pediatric units in hospitals spread across the state of NSW, Australia *(National Public Hospital Establishments Database*). Junior Medical officers (JMOs) frequently rotate through these units and are usually responsible for gentamicin prescribing. While medication prescription is a complex process in children, gentamicin prescription is particularly complicated, as it requires monitoring of the serum levels at specific times and dose adjustment based on the serum levels achieved. Gentamicin has a narrow therapeutic range between efficacy and toxicity; as a result, dosing regimens and adjustments based on serum levels are critical to maintaining optimal efficacy and minimize the risk of toxicity.^11^

Various studies have shown that when standardized care is used quality increases, variation decreases, and cost decreases.^12–14^ A recent major initiative in Australia to improve the safe use of medicines was the introduction of standardized National Inpatient Medication Chart (NIMC).^15^ Despite clear benefits from the standardization of medicines documentation, little is known about the variability in guidelines that support the prescription of gentamicin dosages and drug monitoring practices across different pediatric units and tertiary children’s hospitals in NSW.

We, therefore, aimed to conduct a survey to better understand the practice of gentamicin dosing and therapeutic drug monitoring (TDM) guidelines among various pediatric hospitals and units in the state of NSW.

## METHODS

We developed a multiple-choice knowledge-based questionnaire contingent with our review of gentamicin drug guidelines used at the three tertiary hospitals at NSW: The Sydney Children’s Hospital Randwick, The Children’s Hospital Westmead, and John Hunter hospital (see Appendix 1). This questionnaire was piloted and sent by electronic mail in April 2015 to the Heads of the department at each of the 40 pediatric units across NSW, including the 3 major tertiary children’s hospitals where the survey was sent to a representative pediatric consultant. Neonatal units were excluded from this survey, as another study analyzing gentamicin prescribing practices in the neonatal intensive care units in NSW was underway, during this study period. It was requested that the Heads of the department respond or delegate the responsibility to answer the questionnaire to a JMO, pediatric nurse, or pharmacist from their team. Questions were designed with the purpose that the response options would reflect the unit’s practice on the prescription and TDM of gentamicin for inpatients, based on the guidelines they follow. The survey requested information about the unit’s practice in the following areas.

1. presence of a guideline for gentamicin dosing and TDM;
2. dosing of gentamicin in pediatric patients with normal renal function;
3. dosing interval of gentamicin;
4. maximum daily dosing of gentamicin;
5. methods used for gentamicin TDM;
6. gentamicin dosing and monitoring in febrile neutropenic patients;
7. management of gentamicin drug levels above target range;
8. gentamicin dosing in obese patients;
9. cohort of patients excluded from the unit’s gentamicin guideline.

A reminder electronic mail was sent if no reply was received after 14 days of sending the questionnaire. All responses received were stored in the survey database and transferred to an Excel spreadsheet. The data were descriptively analyzed with frequency described as numbers and percentages. Ethical consent for the study was obtained from Sydney Children’s Hospital Network Human Research Ethics Committee (Reference: LNR.15.SCHN.37).

## RESULTS

A total of 35 of the 40 pediatric units responded to the questionnaire (response rate of 87%). No response was received from 5 regional pediatric units. More than 1 response was received from 8 units, giving a total of 50 responses from 35 units. Where multiple responses were received, the response from the most senior clinician from the unit was selected to represent the unit’s policy.

From the 35 pediatric units, 17 pediatric specialists (48%), 15 pediatric JMOs (43%), 2 pediatric nurses (6%), and 1 pediatric pharmacist (3%) responded to the questionnaire. The majority of the respondents (91%) identified their primary workplace as the pediatric ward in their respective hospitals (Table 1).

**Table 1.**
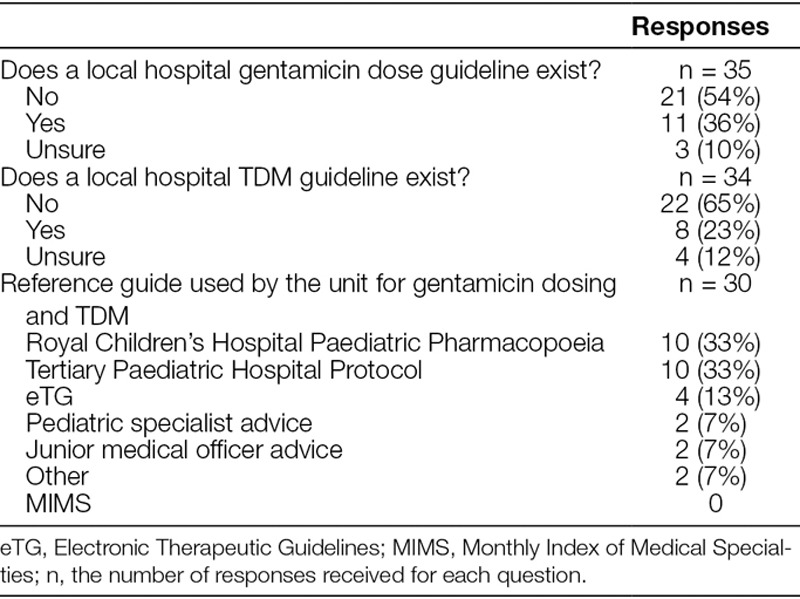
Responses to Questions on Guidelines for Prescription of Gentamicin Medication Used by Clinicians across Various Pediatric Units and Children’s Hospitals in NSW

**Fig. 1.**
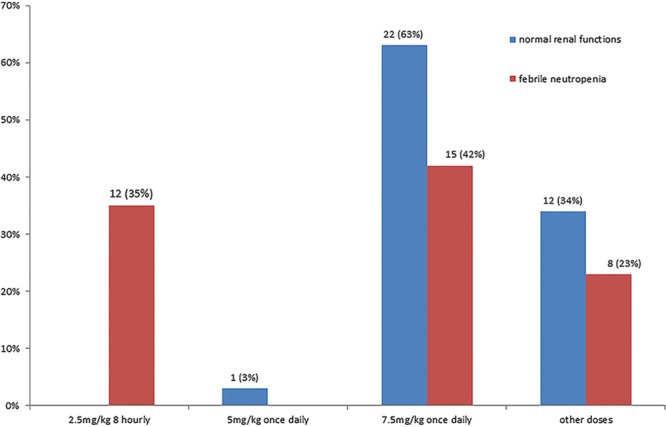
Variation in dosing of gentamicin per kilogram per dose to treat patients with normal renal function and high-risk patients with febrile neutropenia (n = 35).

About half of the respondents (54%) stated that their unit did not have local guidelines for gentamicin dosing and used other information sources, whereas 3 respondents (10%) were unsure if their unit had a guideline for gentamicin dosing. Similarly, for TDM more than half (65%) indicated that they did not have their own local guideline for gentamicin TDM and 4 (12%) respondents stated that they were not aware if a local guideline existed for gentamicin TDM use (Fig. 1).

Most of the respondents (63%) indicated that their practice was to use a single daily dose of 7.5 mg/kg for patients with normal renal function. Forty-two percent of respondents reported the use of the same dose in patients with febrile neutropenia.

Responses coded as “other” were common for the question on the dose of gentamicin used and included age-based dosing responses. Gentamicin dosing responses varied from a dose of 6 mg/kg once daily for patients more than 10 years of age to 7 mg/kg once daily on day 1, followed by 5 mg/kg once daily for patients over 10 years of age.

**Fig. 2.**
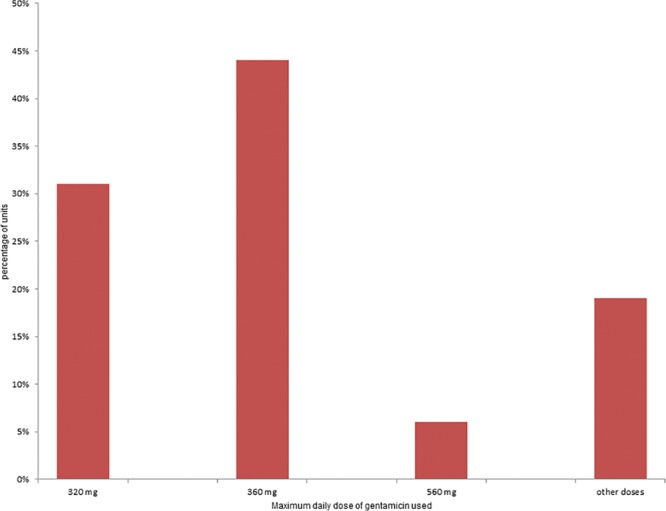
Variability in the maximum dose limit of gentamicin prescribed within 24-hour period (n = 32) among pediatric units and children’s hospitals across NSW.

The dosing regimen used in febrile neutropenia patients varied significantly with approximately a third of units using 2.5 mg/kg 3 times a day, a third using 7.5 mg/kg once daily and a quarter choosing the option indicating another dose (Fig. 2).

There was significant variation in the responses received for maximum dose of gentamicin prescribed in a 24-hour period. The “other” responses option had a wide variation, ranging from the lowest total dose reported as 240 mg to the highest dose reported as 640 mg over a 24-hour period (Table 2).

**Table 2.**
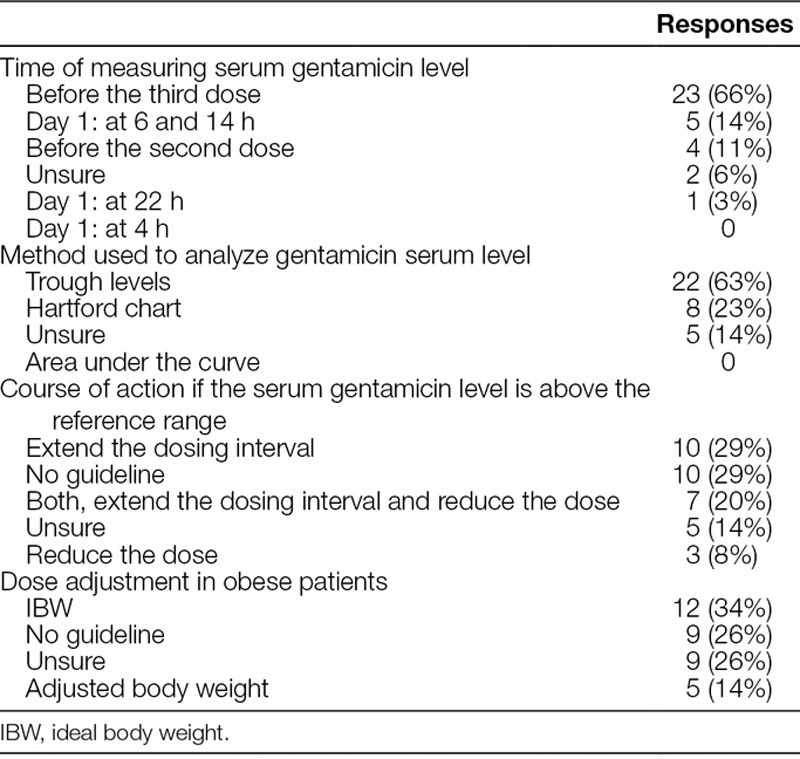
Variations in the TDM of Gentamicin Used by Pediatric units and Children’s Hospitals across NSW (n = 35)

The method used for gentamicin TDM varied markedly, with 63% indicating use of trough levels and 23% using Hartford Nomogram (a nomogram that acts as a guide on when to give the patients their next dose, based on gentamicin concentration and time the measurement was taken). Sixty-six percent of units measure first blood levels for TDM before the third dose, 14% measure it on day 1 at 6 hours, and 11% before the second dose.

**Fig. 3.**
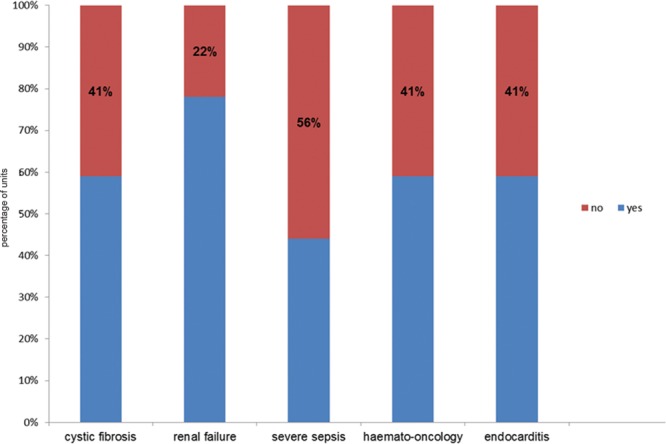
Cohorts of patients excluded from gentamicin prescribing guidelines (n = 32).

A significant proportion (40%) of the respondents were either unsure or had no local guideline on the appropriate course of action if the serum gentamicin levels were high. Similarly, 43% of the respondents were either unsure or had no local guidelines regarding gentamicin dosing for obese patients (Fig. 3).

A significant proportion (44%) of the units indicated that treatment for severe sepsis was excluded in their gentamicin dosing and TDM guidelines, whereas majority (78%) excluded patients with renal failure. Less commonly encountered conditions such as cystic fibrosis and endocarditis were excluded from the guidelines by most units.

## DISCUSSION

Standardization of practice is an important tool in improving quality and patient safety outcomes.^16–18^ It is well known that the adoption of 1 appropriate specific management plan by a clinical care team will, by virtue of standardization alone, yield results superior, to those achieved by random application of several individually equivalent approaches.^19^ Our survey demonstrates the lack of standardization in gentamicin dosing and TDM guidelines among different pediatric units and children’s hospitals in NSW. It presents a significant challenge to clinicians, including JMOs, who must orientate themselves to each new unit’s guideline to prescribe gentamicin. The absence of standardization contributes to confusion in gentamicin prescription and adds to the potential prescription errors related to its administration. This can have direct patient safety effects on young children. To facilitate standardization and consistency of practice, we suggest that a statewide gentamicin dosing and TDM guideline should be developed using a multidisciplinary approach.

Studies in pediatric patients suggest that once daily dosing results in improved clinical efficacy and reduced toxicity compared with multiple daily dosing as the bactericidal activity of aminoglycoside is concentration dependent.^20^ Also, for empirical therapy (<48 hours) monitoring of plasma concentrations is not required.^21^ Although the Australian therapeutics guidelines (2016)^22^ recommend the use of a computerized method with a dosage adjustment to achieve a target AUC, they also realize that not all hospitals would have access to these computer programs. Hence, nomograms were included and the nomograms continue to be used by some units in NSW.

Our survey was answered by more than 1 respondent from 8 pediatric units. Although the numbers of responses received were too small to draw conclusions about intradepartmental variability, a review of the comments section in these responses revealed an element of confusion within some units about gentamicin prescribing guidelines. For instance, in reference to gentamicin drug level monitoring, 1 response stated: “the nursing unit manager and an advanced trainee are in disagreement about the usual practice.” Also, 2 responses stated the gentamicin dose “depends on what source the prescriber uses.” A likely explanation for this discrepancy is the lack of familiarization with the local protocol and the exposure of rotating medical staff to different gentamicin protocols, resulting in intrahospital variation of gentamicin prescribing and monitoring.

The reasons for the lack of standardization of gentamicin dosing and TDM practices across pediatric units in NSW are unclear. It is interesting to note that 60% of children hospitalized in NSW are admitted to “nonpediatric” hospitals.^23^ Our survey demonstrates that many pediatric units follow gentamicin guidelines published by the tertiary children’s hospitals. It is, therefore, confusing that the 3 tertiary children’s hospitals in NSW have variation in guidelines for gentamicin dosing and TDM. Medication guidelines are regularly updated to reflect best practice and recent changes have since been made to these guidelines. However, the absence of a statewide gentamicin guideline remains a significant factor impeding the best practice on gentamicin dosing and its TDM.

No single solution represents a complete answer to this complex issue of gentamicin-related medication errors. Given it is known that a lack of consensus-based guidelines can lead to variation in practice,^24^ this survey suggests that standardization of gentamicin dosing and monitoring guidelines for pediatric patients across all units in NSW has the potential to reduce prescribing errors and prevent drug toxicity. The issue of gentamicin medication errors is not just confined to NSW or Australia but is universal. In the United Kingdom, the NPSA Alert issued in 2010 provides a national care bundle approach with the care bundle’s key elements to include using 24-hour clock, ensuring no interruption during administration, using a double-checking prompt and giving the prescribed dose within 1 hour of the prescribed time.^25^ Gazarian and Graudins^26^ demonstrated a reduction in medication error and harm (specifically paracetamol in hospitalized children in Australia) by using a guideline implementation model whose key components included consensus development, multidisciplinary collaboration, effective clinical leadership, interactive education, and timely data feedback using iterative Plan–Do–Study–Act cycles. Similar approaches, specifically targeting gentamicin prescription, should be explored in pediatric units and children’s hospitals in NSW.

There has been growing interest in the use of computerized physician order entry system as a potential solution to reduce medication errors across hospitals in NSW. Despite benefits in improving quality of medication orders in terms of legibility and completeness, limitations to its use exist such as variable ease of use, physician acceptance, cost, software integration into existing facility systems, standardization across systems, potential increase in errors after implementation, and ability to address only a subset of potential medical errors.^27^ The use of a standardized, easy to use gentamicin-specific medication chart deserves special consideration. Standardized individualized medication charts can lead to reductions in prescribing errors and result in improved documentation.^28^ Three previous studies in Australia, where standardized charts were used to improve management of particular pharmacological therapies (insulin, venous thromboembolism prophylaxis, warfarin, and N-acetylcysteine), demonstrated improvements in prescribing practice.^29–31^ A study in a neonatal unit in Northern Ireland reported 16% reduction in the average number of gentamicin-related medication errors since the introduction of a gentamicin-specific drug chart in their unit.^32^ Pharmacists play an important role in medication management. In the United States, 95% of hospitals have pharmacists monitor serum medication concentrations and most of these hospitals allow pharmacists order concentrations and make adjustments in the dose.^33^ We advocate for a greater utilization of pharmacists in gentamicin drug monitoring and prospective review of all gentamicin dose prescriptions.

We acknowledge that our review of gentamicin prescription practices is subject to some limitations. The survey questions were designed with their multiple-choice response options to reflect on the unit’s clinical practice on dosing and monitoring of gentamicin. However, we acknowledge the fact that it is possible that the responses may have been biased by individual clinician practice opinions. We did not individually collect each local hospital approved guideline on gentamicin dosing and TDM as we wanted to examine the knowledge and practical applications of these guidelines based on the staff responses. Also, nurses and pharmacists play an important role in coordinating all aspects of the implementation of gentamicin administration guidelines and their participation in this survey was limited. It is therefore with some caution that we make generalizations about the current prescribing practices in NSW, but we recognize that our results, in general, reflect the clinical practice of the medical practitioner responsible for gentamicin prescription.

The conclusions from our study provide a strong basis for an improvement collaborative such as to develop a simple and standardized approach to gentamicin dosing and its monitoring for all pediatric units in NSW. There is an urgent need to develop consistent, accurate, and reliable guidelines for calculating the dose and TDM protocols for gentamicin across all pediatric hospitals in the state. Options include development of a standardized paper or electronic specific gentamicin prescription chart for use in all pediatric units. The options to consider in such a form, either paper or electronic based, could be that the dose of gentamicin be checked at the time of writing by 2 clinicians and all gentamicin prescribing charts be reviewed by a pharmacist within 24 hours of prescribing. We suggest that a protocol such as that outlined above has a potential for a significant reduction in gentamicin-related adverse events and should be implemented and evaluated with a post-introduction audit.

## ACKNOWLEDGMENTS

The authors would like to thank all the pediatricians, junior medical officers, nurses, and pharmacists who participated in this survey. We are grateful to Professor Les White, chief pediatrician, New South Wales, for his help and support in the distribution of this survey. We would also like to thank Paul Hunstead, Project Officer, Clinical Excellence Commission for providing us with data on gentamicin-related medication errors from the state of New South Wales.

## DISCLOSURE

The authors have no financial interest to declare in relation to the content of this article.

## APPENDIX 1

### The questionnaire requested the representative to answer the following questions:

1. Does your unit have its own hospital guideline for gentamicin dosing and TDM for pediatric patients (aged 1 month to 16 years)?
2. If no, what reference source does your unit usually use for gentamicin dosing and TDM?
3. What intravenous dose of gentamicin does your organization use in the age group 1 month to 16 years for children with normal renal function and who have been previously well?
4. What is the maximum gentamicin dose that your organization uses?
5. If a child (aged 1 month to 16 years) with febrile neutropenia with normal renal function presents to your hospital needing gentamicin, what dosing of gentamicin does your organization recommend?
6. What approach does your organization use to monitor gentamicin levels?
7. For a child (aged 1 month to 16 years) on prolonged directed therapy (>48 hours), at what time point during gentamicin treatment does your organization recommend taking the first blood levels for TDM for patients who were previously well?
8. In the case of gentamicin serum levels of TDM being high, which of the following actions would you follow? Options included: extend the dosing interval, reduce the gentamicin dose, both extend the dosing interval and reduce the gentamicin dose, unsure, and no guideline exists.
9. In the case of obese patients, how does your organization determine the dose of gentamicin to be administered?
10. Which cohort of patients are excluded in your guidelines on gentamicin dosing and TDM? Options included: cystic fibrosis, renal failure, severe sepsis, hematology oncology patients, and patients with endocarditis.

## References

[1] Avent ML Rogers BA Cheng AC Current use of aminoglycosides: indications, pharmacokinetics and monitoring for toxicity. Int Med J. 2011;41(6):441–449. doi: 10.1111/j.1445-5994.2011.02452. 10.1111/j.1445-5994.2011.02452.x 21309997

[2] Bates DW Medication errors. Drug Safety. 1996;15(5):303–310. doi: 10.2165/00002018-199615050-00001. 8941492 10.2165/00002018-199615050-00001

[3] http://NationalPatient Safety Agency (2010). The safer use of intravenous gentamicin for neonates. http://www.nrls.npsa.uk/resources/type/alerts/?entryid45=66271&q=0%c2%acgentamicin%c2%ac. Accessed January 2, 2017.

[4] Metsvaht T Nellis G Varendi H High variability in the dosing of commonly used antibiotics revealed by a Europe-wide point prevalence study: implications for research and dissemination. BMC Pediatrics. 2015;15(1):41 doi: 10.1186/s12887-015-0359. 25880733 10.1186/s12887-015-0359-y PMC4407781

[5] Newham R Thomson AH Semple Y Barriers to the safe and effective use of intravenous gentamicin and vancomycin in Scottish hospitals, and strategies for quality improvement. Eur J Hosp Pharm. 2014;22(1):32–37. doi: 10.1136/ejhpharm-2014-000483.

[6] Feleke Y Girma B Medication administration errors involving paediatric in-patients in a hospital in Ethiopia. Trop J Pharmaceut Res. 2010;9(4):401–407. doi: 10.4314/tjpr.v9i4.58942.

[7] Rogers MS Improved compliance with a gentamicin prescribing policy after introduction of a monitoring form. J Antimicrob Chemother. 2005;56(3):566–568. doi: 10.1093/jac/dki279. 16076879 10.1093/jac/dki279

[8] Egan S Murphy PG Fennell JP Using Six Sigma to improve once daily gentamicin dosing and therapeutic drug monitoring performance. BMJ Qual Safety. 2012;21(12):1042–1051. doi: 10.1136/bmjqs-2012-000824. 10.1136/bmjqs-2012-000824 22871475

[9] Qureshi DI Habayeb MH Grundy DC Improving the correct prescription and dosage of gentamicin. BMJ Qual Improv Rep. 2012;1(1):u134–w317. doi: 10.1136/bmjquality.u134.w317. 10.1136/bmjquality.u134.w317 PMC4652673 26734148

[10] Wong E Taylor Z Thompson J A simplified gentamicin dosing chart is quicker and more accurate for nurse verification than the BNFc. Arch Dis Child. 2008;94(7):542–545. doi: 10.1136/adc.2007.137026. 18676437 10.1136/adc.2007.137026

[11] Roberts JA Norris R Paterson DL Therapeutic drug monitoring of antimicrobials. Br J Clin Pharmacol. 2011;73(1):27–36. doi: 10.1111/j.1365-2125.2011.04080. 10.1111/j.1365-2125.2011.04080.x PMC3248253 21831196

[12] Darmstadt GL Bhutta ZA Cousens S Evidence-based, cost-effective interventions: How many newborn babies can we save? Lancet. 2005;365(9463):977–988. doi: 10.1016/s0140-6736(05)71088-6. 15767001 10.1016/S0140-6736(05)71088-6

[13] Landon BE Normand S-LT Blumenthal D Physician clinical performance assessment. JAMA. 2003;290(9):1183 doi: 10.1001/jama.290.9.1183. 12953001 10.1001/jama.290.9.1183

[14] Timmermans S Mauck A The promises and pitfalls of evidence-based medicine. Health Aff. 2005;24(1):18–28. doi: 10.1377/hlthaff.24.1.18. 10.1377/hlthaff.24.1.18 15647212

[15] Coombes ID Reid C McDougall D Pilot of a national inpatient medication chart in Australia: Improving prescribing safety and enabling prescribing training. Br J Clin Pharmacol. 2011;72(2):338–349. doi: 10.1111/j.1365-2125.2011.03967. 21426371 10.1111/j.1365-2125.2011.03967.x PMC3162663

[16] Rycroft-Malone J Fontenla M Seers K Protocol-based care: The standardisation of decision-making? J Clin Nurs. 2009;18(10):1490–1500. doi: 10.1111/j.1365-2702.2008.02605. 19413539 10.1111/j.1365-2702.2008.02605.x

[17] Sunde K Pytte M Jacobsen D Implementation of a standardised treatment protocol for post resuscitation care after out-of-hospital cardiac arrest. Resuscitation. 2007;73(1):29–39. doi: 10.1016/j.resuscitation.2006.08.016. 17258378 10.1016/j.resuscitation.2006.08.016

[18] Wood DL Brennan MD Chaudhry R Standardized care processes to improve quality and safety of patient care in a large academic practice: The Plummer project of the Department of Medicine, Mayo clinic. Health Serv Manage Res. 2008;21(4):276–280. doi: 10.1258/hsmr.2008.008009. 18957404 10.1258/hsmr.2008.008009

[19] Clark SL Nageotte MP Garite TJ Intrapartum management of category II fetal heart rate tracings. Obstet Anesth Digest. 2014;34(3):132–133. doi: 10.1097/01.aoa.0000452144.12033. 10.1016/j.ajog.2013.04.030 23628263

[20] Bass KD Larkin SE Paap C Pharmacokinetics of once-daily gentamicin dosing in pediatric patients. J Pediatr Surg. 1998;33(7):1104–1107. 9694103 10.1016/s0022-3468(98)90540-1

[21] Lee J Yoon S Shin D Predictive performance of gentamicin dosing nomograms. Drug Des Devel Ther. 2014;8:1097. 10.2147/DDDT.S66981 PMC4140707 25152616

[22] Alam M Bastakoti B Therapeutic guidelines: Antibiotic. Version 15. Australian Prescriber. 2015;38(4):137–137. doi: 10.18773/austprescr.2015.049.

[23] Population health data warehouse. http://www.health.nsw.gov.au/epidemiology/Pages/Population-health-data-warehouse.aspx. Accessed January 2, 2017.

[24] Woolf SH Grol R Hutchinson A Clinical guidelines: Potential benefits, limitations, and harms of clinical guidelines. BMJ. 1999;318(7182):527–530. doi: 10.1136/bmj.318.7182.527. 10024268 10.1136/bmj.318.7182.527 PMC1114973

[25] Upton M Gooding N Ashmore R A regional approach to implementation of the NPSA gentamicin alert. Infant. 2012;8:14–19.

[26] Gazarian M Graudins LV Long-term reduction in adverse drug events: An evidence-based improvement model. Pediatrics. 2012;129(5):e1334–e1342. doi: 10.1542/peds.2011-190. 22473370 10.1542/peds.2011-1902

[27] Takata Principles of patient safety: Reducing harm due to medical care. Pediatrics. Pediatrics. 2011;2011;127(128(6):6):1199–1212–1210. 1212. doi: 10.1542/peds.2011-1758. 21624879 10.1542/peds.2011-0967

[28] Roughead L Semple S Rosenfeld E Literature Review: Medication Safety in Australia. 2013 Sydney: Australian Commission on safety and Quality in Health Care;

[29] McIver FB Mitchell CA Finn CP Standardising practices through form design and education improves insulin management. Austral Health Rev. 2009;33(3):434 doi: 10.1071/ah090434. 10.1071/ah090434 20128759

[30] Liu DSH Lee MMW Spelman T Medication chart intervention improves inpatient thromboembolism prophylaxis. Chest. 2012;141(3):632–641. doi: 10.1378/chest.10–3162. 21778254 10.1378/chest.10-3162

[31] McIntyre S Greene S McD Taylor D Response to introduction of an N-acetylcysteine weight-based dosing chart reduces prescription errors in the treatment of paracetamol poisoning: Reply. Emerg Med Australasia. 2013;25(3):286–287. doi: 10.1111/1742–6723.12084. 10.1111/1742-6723.12084 23759056

[32] Flannigan C Kilpatrick S Redpath J Can a gentamicin-specific chart reduce neonatal medication errors? Clin Audit. 2010;2010:7–11. doi: 10.2147/ca.s8424.

[33] Pedersen CA Schneider PJ Scheckelhoff DJ ASHP national survey of pharmacy practice in hospital settings: Monitoring and patient education–2015. Am J Health-System Pharm. 2016;73(17):1307–1330. doi: 10.2146/ajhp160081. 10.2146/ajhp160081 27413141

